# Does Adding Social Cognitive Remediation Therapy to Neurocognitive Remediation Therapy Improve Outcomes in Young People With a Severe Mental Illness?—The Advantage Trial

**DOI:** 10.3389/fpsyt.2021.789628

**Published:** 2022-03-14

**Authors:** Anthony W. F. Harris, Michelle Kightley, Joanna Williams, Cassandra Ma, Carlie Dodds

**Affiliations:** ^1^Specialty of Psychiatry, University of Sydney School of Medicine, The University of Sydney, Sydney, NSW, Australia; ^2^Brain Dynamics Centre, The Westmead Institute for Medical Research, The University of Sydney, Sydney, NSW, Australia; ^3^Child and Youth Mental Health Service, Western Sydney Local Health District, North Parramatta, NSW, Australia

**Keywords:** cognitive remediation, social cognition, schizophrenia, social cognition rehabilitation, bipolar disorder, youth mental health, outcome, community function

## Abstract

**Introduction:**

Cognitive impairments are a common and significant issue for young people with a severe mental illness. Young people with schizophrenia, bipolar disorder and major depression all experience significant cognitive problems that impede their ability to return to work or study. These neurocognitive problems are frequently exacerbated by social cognitive deficits that interfere with their ability to integrate into the community and understand the social and emotional nuances about them. This study aimed to assess if the addition of a social cognitive remediation treatment to a neurocognitive remediation therapy improved functional outcome.

**Methods:**

Five youth mental health services were trained in both the Neuropsychological Educational Approach to Remediation (NEAR) and the Social Cognition and Interaction Training (SCIT) treatments. Participants were randomised between receiving either NEAR + SCIT or NEAR + treatment as usual (TAU) over a 20-week period, with all participants receiving the NEAR treatment first. Symptoms, neurocognition, social cognition and functioning were examined at baseline, end of treatment and at 3 months follow-up and compared between the two arms of the study. The primary outcome was function.

**Results:**

Thirty-nine participants were randomised to treatment (Schizophrenia spectrum = 28, Bipolar disorder = 7, Major Depression = 2). The trial was curtailed by Covid-related service restrictions. There was an overall significant improvement in function over time with a trend towards a greater improvement in the NEAR + SCIT arm. No changes in symptoms, neurocognitive or social cognitive measures were seen. While 74% completed treatment only 49% agreed to follow up at 3 months affecting our ability to interpret the findings. Attrition did not differ by arm.

**Conclusions:**

In a pragmatic, service-based research project, treatment aimed at improving cognition enhanced functional outcome in young people with a range of severe mental illnesses. There was a trend towards improved function in young people who had a combined NEAR + SCIT approach.

**Clinical Trial Registration:**

Identifier: ACTRN12622000192785.

## Introduction

Remission of psychotic symptoms is a common primary treatment metric used to assess treatment response in severe mental illnesses (SMI) such as schizophrenia, schizophreniform disorder or bipolar disorder, however functional recovery, a more important but frequently secondary outcome, remains elusive for many. A significant contributor to this failure to achieve functional recovery is the detrimental effects of neurocognitive and social cognitive deficits in SMI. Initially, investigations of neurocognitive and social cognitive deficits centred upon schizophrenia (1–3), however there is now a more holistic approach that these deficits are common to all SMI (4), albeit with different degrees of severity.

Neurocognition and social cognition are important because of their contribution to the ability of the individual to operate in a complex society, however their effects appear to be different though overlapping (5). While neurocognitive deficits are basic to the impact of cognition on function, both due to a direct effect and *via* its influence on social cognition, social cognition may have a greater effect on community functioning overall both by its direct effect and via moderating neurocognitive deficits (6). This suggests that targeting neurocognitive deficits alone is not sufficient to improve overall outcome. Unfortunately, the cognitive deficits in SMI are not ameliorated by standard antipsychotic therapies (7). This along with the recognition of the important role played by cognitive deficits in the outcome of SMI (6) has generated new treatment approaches such as cognitive remediation therapy (CRT) and more recently, social cognitive remediation therapy (SCRT). These treatments have a small to moderate effect upon cognitive function and this is noted to generalise to functional improvement (8, 9). This positive effect on functional recovery is helped by combining cognitive treatments with other psychosocial approaches such as supported employment (10), however, the effectiveness of combining the two approaches to cognitive remediation—therapy for neurocognitive as well as social cognitive deficits—is less frequently investigated. This is surprising given that neurocognition, at least in some analyses, appears to be a foundation for the mediating effect of social cognition upon eventual community functioning (6, 11, 12).

Of the studies that have examined the usefulness of combining neurocognitive and social cognitive remediation strategies, most have been in chronically unwell, predominantly male participants (13–16). While CRT clearly improved neurocognition (8), this effect was enhanced by the combination of neurocognitive and social cognitive remediation therapies (15). But improvement in social cognition required the specific treatment of that domain (13, 16). Treatment of cognition translated into better community function (15), though whether the combination of both treatments is necessary for this effect is not clear (13, 16). This study aimed to test the effectiveness of combining CRT with SCRT against CRT alone in improving community functioning in a group of young people with severe mental illness. In addition, it followed participants up over 3 months to see if any improvements were maintained longer term. We predicted that the combined treatment of neurocognitive and social cognitive remediation would have a superior effect on functional outcome over neurocognitive remediation alone.

## Method

This trial was a single blind randomised controlled trial conducted in five youth mental health services across Sydney, Australia. All participants were aged between 17 and 25 years of age; had a diagnosis of a severe mental illness (first episode psychosis, schizophrenia, schizophreniform disorder, bipolar disorder, or major depressive disorder); neurocognitive or social cognitive deficits; were able to provide consent (and parent/guardian if required); and had reasonable English skills. Participants were excluded if they had a developmental delay (IQ <75); current substance abuse or substance dependence other than caffeine or nicotine; a history of head injury (> 10 min unconsciousness); or had been treated with electroconvulsive therapy in the last 6 months.

Participants were randomised between two arms on a 1:1 ratio. Participants in the treatment arm were provided with a combination of Cognitive Remediation Therapy [using Neuropsychological Educational Approach to Remediation or NEAR (17)] and social cognitive remediation (using Social Cognition and Interaction Therapy or SCIT (18). This was compared to a control arm of CRT (NEAR) + the additional treatment available at the service where the treatment was provided.

NEAR (17) is a manualised CRT designed to address cognitive deficits by utilising commercially available educational software to create a rich learning environment that is intrinsically motivating and rewarding. The treatment was provided over 10 weeks, two times per week to participants in groups averaging four people. All participants received NEAR.

SCIT (18) is a manualised treatment designed to address social cognitive deficits. It consisted of 20 1-h sessions over 10 weeks. Training was run in small groups of three to six people using a manual-driven suite of activities. The training approach of SCIT is such that participants receive repeated exposure and practise of the skills that underlie complex mental-state reasoning abilities. The CRT-only group had a range of additional active comparator treatments including physical exercise, social skills groups, individual therapy, or no additional treatment. Both arms received the same duration of treatment-−20 weeks of twice-weekly treatment. All therapists were trained and supervised for the duration of the study, however adherence to the manualised treatments was not formally audited.

Participants were randomised in blocks of four participants from a central register that was operated by administrative staff independent to the services involved. The randomisation sequence was stratified by site and was generated using an online randomisation generator (Sealed Envelope https://www.sealedenvelope.com/simple-randomiser/v1/lists) by a statistician affiliated with the University of Sydney and independent to the research team. Allocation was blind to the research psychologist performing all cognitive and clinical assessments. Allocation was revealed to the treating clinical team by the administrative staff after consent had been obtained and baseline measures taken.

Participants were assessed at baseline, at the completion of treatment and 3 months following the completion of treatment, on a broad range of clinical, cognitive, and functional measures by a psychologist blind to allocation. Initial demographic and clinical details was collected using a semi-structured interview that detailed age, duration of illness, age of onset of illness, treatment history, medication dose, years of education, past employment history, relationship history. Clinical psychopathology was rated using the Positive and Negative Syndrome Scale-−6 items (PANSS-6) (19), the Calgary Depression Scale for Schizophrenia (CDSS) (20) and the Depression Anxiety and Stress Scale (DASS-21) (21).

Neuropsychological Function was assessed using a battery of neuropsychological measures to assess aspects of attention, concentration, vigilance, verbal learning, executive functioning, planning and premorbid intelligence including The two-subtest versions of the Wechsler Abbreviated Scale of Intelligence (WASI) was used to provide an estimate of IQ (22). The two subtests include Vocabulary and Matrix Reasoning. The Repeatable Battery for the Assessment of Neuropsychological Status (RBANS) assesses five indexes of neurocognition such as immediate memory, delayed memory, attention, construction visuospatial and language (23). Information processing speed and attentional control were assessed with the Trail-Making Test Part A and B. Social cognition was assessed using the Hinting Task (24) as a measure of Theory of Mind (ToM), the Penn Emotion Recognition Test (ER40) (25) as a test of emotion recognition; and the Ambiguous Intentions Hostility Questionnaire (AIHQ-A) as a test of attributional style (26).

Community functioning was assessed using the Social and Occupational Functioning Assessment Scale (SOFAS), which is an interviewer rated scale based on the evaluation of the participants' social and occupational functioning (27) and via the Activity and Participation Questionnaire (APQ) (28). The Assessment of Quality of Life (AQoL-8D) was used to measure subjective satisfaction and well-being (29).

### Statistical Analysis

Participants were assessed on the battery of neurocognitive and functional measures at baseline, post-treatment, and follow-up at 3 months after the treatment. Repeated measures analysis of variance (ANOVA) was used to assess any potential interactions across time and between treatment groups. A chi-squared analysis was conducted to compare the demographics between the treatment and control groups inclusive of the diagnosed SMI and medication. Statistical analyses were conducted using SPSS Statistical Packaging for the Social Sciences (30).

## Results

**Figure 1:**
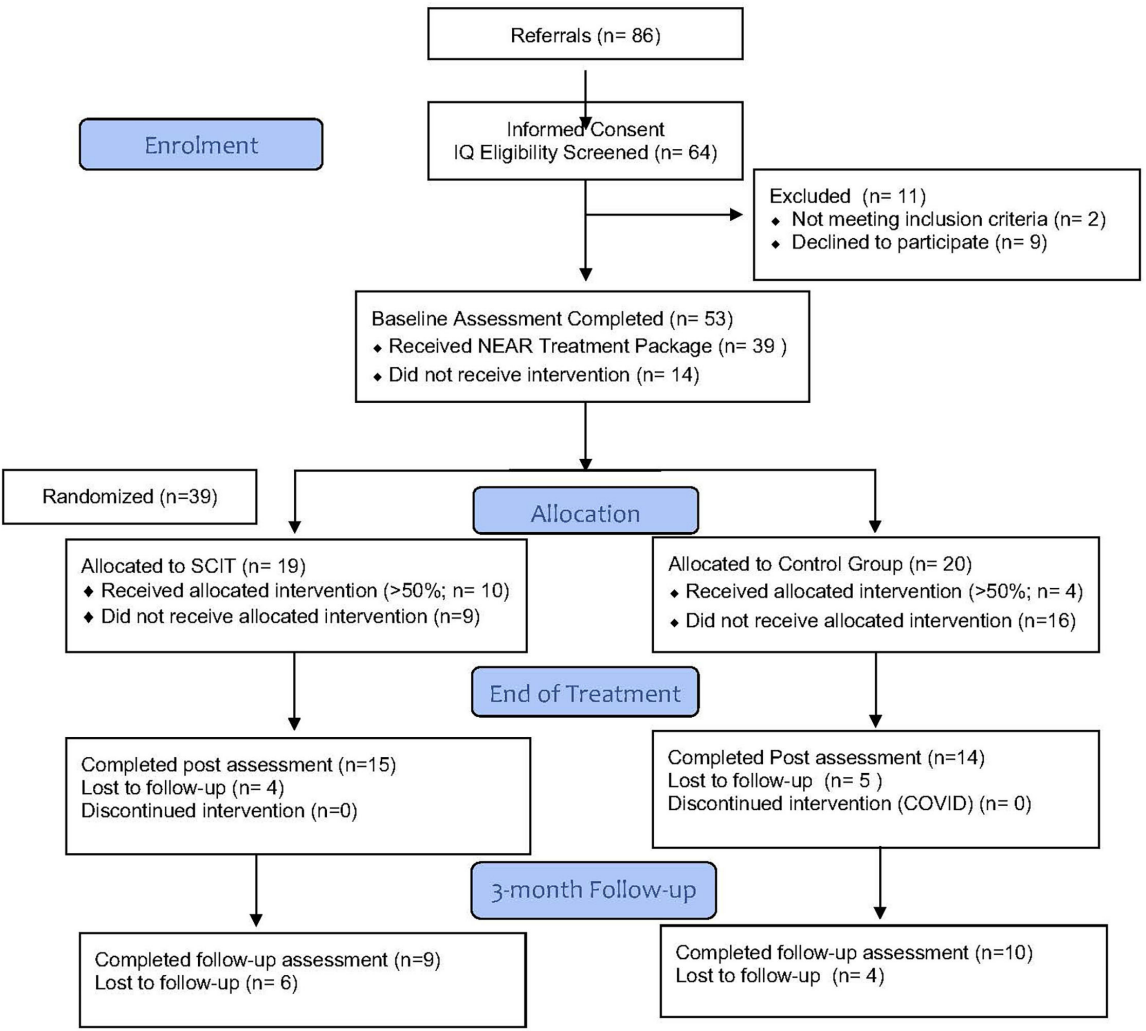
CONSORT Diagram of the Advantage Trial.

A total of 39 participants were randomised between the two arms of the study (see Figure 1). The study group had an average age of 21.7 yrs (sd 3.0 yrs) and a duration of illness of 3.1 yrs (sd 2.5 yrs). Sixty five percentage of the group were male. There were no significant differences between treatment and control groups based on gender, diagnosis of SMI, medication, education, previous employment, and relationship status (see Table 1). It should be noted that percentages for medication does not add up to 100% due to some participants taking multiple medications, which is clinically common. Drop out through the trial was high with only 49% of participants completing the 3-month follow-up. There was no difference in participant attrition between the arms.

**Table 1:** Demographic variables.

	**Control *N* (%)**	**Treatment *N* (%)**	***p***
**Gender**			0.109
Female	4 (10.8)	9 (24.3)	
Male	14 (37.8)	10 (27.0)	
**Diagnosis**			0.299
Schizophrenia	5 (13.5)	8 (21.6)	
First episode psychosis	6 (16.2)	7 (18.9)	
Schizoaffective disorder	2 (5.4)	0 (0)	
Bipolar affective disorder	3 (8.1)	4 (10.8)	
Major depressive disorder	2 (5.4)	0 (0)	
Other	0 (0)	0 (0)	
Medication^*^			
No medication	–	–	
Antipsychotic	14 (37.8)	16 (43.2)	0.618
Antidepressant	6 (16.2)	8 (21.6)	0.582
Mood stabiliser	4 (10.8)	5 (13.5)	0.772
Other	1 (2.7)	2 (5.4)	0.580
**Chlorpromazine equivalent (M, sd)**	377.77 (295.40)	227.92 (177.04)	0.187
**Education**			
Year 10 equivalent	18 (48.6)	19 (51.4)	–
HSC equivalent	16 (43.2)	12 (32.4)	0.068
University training	6 (16.7)	6 (16.7)	0.638
Tertiary training	8 (22.2)	6 (16.7)	0.342
**Previous employment**	13 (35.1)	12 (32.4)	0.556
**Relationship**			0.079
Single	18 (48.6)	16 (43.2)	
Dating	0 (0)	3 (8.1)	

* *Percentages don't add to 100% due to participants taking multiple medications*.

**Figure 2:**
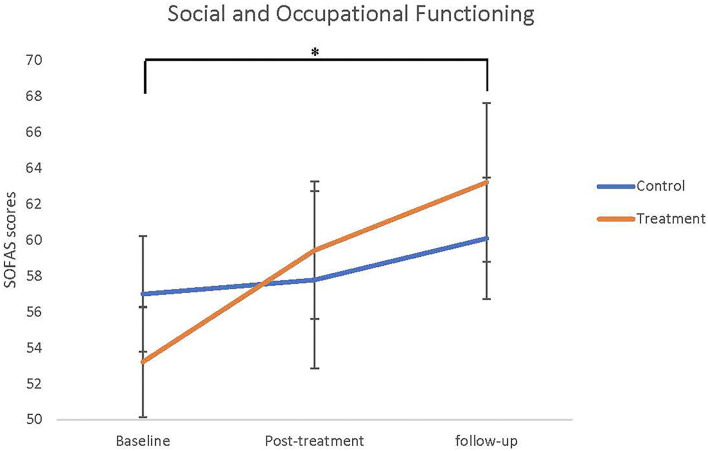
Social and Occupational Functioning Assessment Scale (SOFAS) scores means (SEMs) as baseline, post-treatment, and follow-up. Asterisk indicated significant main effect of time from baseline to follow-up, but there were no differences between groups and interaction.

The neurocognitive and functional outcomes at baseline, post-treatment and follow-up for the treatment and control groups are summarised in Table 2. Repeated measures ANOVA indicated that there was a significant main effect of time from baseline to post-treatment on the hinting task (*F* = 8.880, *df* = 2, *p* < 0.001). Pairwise comparisons adjusted for multiple comparisons revealed that this effect was only significant from baseline to follow-up (*p* = 0.005), and was not significant when measuring from baseline to post-treatment (*p* = 0.073), or post-treatment to follow-up (*p* = 0.160). There was no significant difference between groups and no interaction effect. Similarly, there was a significant main effect of time for the SOFAS measure from baseline to post-treatment (*F* = 5.500, *df* = 2, *p* = 0.009), as can be seen from Figure 2. Again, pairwise comparisons showed that this effect was only significant when comparing baseline to follow-up (*p* = 0.010), and was not significant when comparing baseline to post-treatment (*p* = 0.480) or post-treatment to follow-up (*p* = 0.183), and there was no significance between groups and no interaction effect. There were no significant differences between groups or over time for any other neurocognitive, clinical, or functional measures (all *p* > 0.05).

**Table 2:** Results: Neurocognitive and social functioning scales means and standard errors at baseline, post-treatment and follow-up for the control and treatment arms.

		**Control**			**Treatment**			**Group**		**Time**		**Interaction**	
**Measure**	***n***	**Baseline**	**Post-treatment**	**Follow-up**	**Baseline**	**Post-treatment**	**Follow-up**	**F**	***p***	**F**	***p***	**F**	***p***
**Cognitive**													
RBANS	19	76.8 (5.11)	80.1 (5.48)	77.3 (6.23)	75.67 (3.72)	76.44 (4.60)	81.33 (3.44)	0.002	0.967	0.457	0.637	0.714	0.497
Hostility Bias	18	10.22 (0.72)	10.78 (1.37)	10.44 (0.94)	12.56 (1.81)	11.44 (1.75)	10.89 (1.60)	0.372	0.550	0.629	0.540	1.262	0.297
Intentionality Bias	19	16.7 (1.42)	16.6 (1.94)	16.6 (1.80)	17.33 (2.01)	16.78 (1.61)	15.89 (1.94)	0.000	0.989	0.362	0.699	0.282	0.756
Anger Score	19	11.6 (1.44)	12.7 (1.78)	13.4 (1.56)	11.78 (1.22)	12.56 (1.36)	10.44 (1.68)	0.251	0.623	0.699	0.504	2.171	0.130
Blame Score	19	11.4 (1.13)	12.8 (1.45)	12.7 (1.33)	12.56 (1.71)	13.44 (1.17)	10.78 (0.94)	0.001	0.979	1.321	0.280	1.643	0.208
Aggression Bias	18	8.11 (0.59)	8.44 (0.47)	9 (0.5)	7.89 (0.54)	9.33 (0.88)	9.33 (0.5)	0.382	0.545	2.346	0.112	0.487	0.619
ER40 Correct Responses	19	30.8 (1.14)	31.5 (1.39)	31.6 (1.28)	31.89 (1.27)	33 (1.15)	33.22 (1.23)	0.812	0.380	1.245	0.301	0.074	0.929
ER40 Response Time (ms)	19	2479.45 (226.33)	2353.2 (332.05)	2589.5 (192.98)	2318.56 (190.05)	2058.22 (303.81)	2523.78 (267.70)	0.375	0.549	1.555	0.226	0.167	0.847
Hinting Score	19	13.8 (1.60)	14.9 (1.42)	15.6 (0.89)	13.33 (0.80)	15.22 (1.06)	17.11 (0.63)	0.101	0.754	8.880	<0.001	1.130	0.335
**Clinical**													
DASS	12	19 (7.7)	18.67 (6.32)	20 (6.95)	29.33 (3.57)	24.5 (5.62)	22.17 (6.36)	0.588	0.461	0.522	0.601	0.798	0.464
PANSS-6	19	14.5 (1.69)	14.4 (1.57)	14.4 (1.77)	14.78 (2.13)	13.78 (1.93)	12.22 (1.74)	0.124	0.730	1.477	0.243	1.281	0.291
CDSS	19	6.3 (1.65)	5.9 (1.7)	6 (1.84)	7.56 (2.37)	6.78 (2.09)	4.89 (1.55)	0.021	0.887	1.013	0.374	0.734	0.487
**Functional**													
AQoL	19	89.1 (8.09)	87.6 (8.03)	85.1 (7.63)	91.33 (5.69)	91.11 (6.03)	86.11 (6.96)	0.055	0.818	1.334	0.277	0.087	0.917
APQ hours	18	17.11 (3.61)	16.33 (2.52)	15.67 (4.19)	18.13 (3.61)	14.77 (3.20)	11.67 (1.67)	0.114	0.740	0.474	0.627	0.191	0.827
SOFAS	19	57 (3.06)	57.8 (3.81)	60.1 (4.42)	53.22 (3.22)	59.44 (4.92)	63.22 (3.37)	0.004	0.948	5.500	0.009	1.689	0.200

*Between (group), within (time) and interaction effects are reported from a repeated measures analysis of variance. RBANS, Repeatable Battery for the Assessment of Neuropsychological Status; ER40, Penn Emotion Recognition Test; DASS, Depression Anxiety and Stress Scale; PANSS-6, Positive and Negative Syndrome Scale-6; CDSS, Calgary Depression Scale for Schizophrenia; AQoL, Assessment of Quality of Life-8D; APQ, Activity and Participation Questionnaire; SOFAS, Social and Occupational Functioning Assessment Scale*.

## Discussion

This study observed a positive effect on functioning in young people whether they received NEAR alone or NEAR and SCIT together. There was a weak trend suggesting that the combination of NEAR and SCIT improved functioning at 3 months follow-up. This finding is consistent with previous studies that found an improvement in functioning with exposure to both CRT and SCRT (16, 31, 32), with the suggestion that a broader based approach to remediation is more likely to lead to improvements in community functioning (9). The young people who persisted in therapy in the Advantage treatment group had a change of 10 SOFAS points as against 3 points for active control group over the nearly 9 months. This is a clinically significant improvement (33). Other studies have had mixed results with some finding a greater propensity to change in younger participants, such as ours compared to older, more chronically unwell people (34) or noting little (35) or no change in community functioning (36).

In contrast to our expectations, there were no significant differences in neurocognition or social cognition between groups or across time in either treatment arm. A possible explanation is that the RBANS was not sensitive to change in this group of young people that were less chronically unwell and relatively better educated. However, the group scores indicated a moderate level of neurocognitive impairment and ceiling effects are not a reason for the lack of change (37). The lack of change in social cognitive scores was also surprising. The lack of significant improvement in cognition accompanying an improvement in function was noted by Revell in her meta-analysis of cognitive remediation in early schizophrenia which found that higher quality blinded studies did not observe as much change as non-blinded studies (38). In our study the rater was blind to assignment. Another possibility is that young people may be less able to benefit from intensive training on computer assisted cognitive remediation compared to older and more chronically ill participants as they have more recently been exposed to teaching and training. Our sample was young and comparatively well-educated. We also note that the diagnostic heterogeneity of our sample with several subjects with affective disorders may have lessened the effect size of the cognitive improvement.

Participant attrition was high. Our participants were involved in two sequential treatment programs of 10 weeks duration followed by a further 3-month follow-up, totalling a commitment of nearly 9 months with the breaks required for testing. The biggest dropout rate was during the second phase of the study, when participants received either SCIT or TAU and after during follow-up. This was consistent across sites and suggests treatment fatigue among the young people coming for treatment. We note that high rates of participant attrition is not uncommon amongst long-term interventional studies. For example, Fisher and colleagues' (16) attrition loss of 45% of their participants over 33 weeks training was despite compensation for their attendance. In a randomised controlled trial of cognitive enhancement therapy in young people, Wojtalik et al. lost 52% of their sample over 18 months of treatment (35). This is in accordance with our own experience and that of the literature, that treatment programs limited to 12–16 weeks have a lower attrition rate. For example, Vidarsdottir et al. (36) combined NEAR, SCIT and additional compensatory cognitive training into a more intense 12 week program that in content was similar to our program and in a very similar group of young people. Their participant attrition rate was only 12%.

Although our study was not conclusive, it does support the importance of addressing social cognitive deficits as well as neurocognitive deficits in people with severe mental illness to improve community outcomes. Recovery in people with severe mental illness like schizophrenia has not improved despite the availability of new medications and psychotherapies (39). Recent large studies have underlined the contribution that social and neurocognition make to community function and the complex interaction at play with psychopathology—both negative symptoms and positive symptoms (40–42). The use of targeted cognitive remediation strategies early in the course of illness may help improve the long-term outcome of people with severe mental illness. Further work is required to explore the dynamics of how this is achieved.

Our study has several limitations. It was curtailed by the start of the global Covid-19 pandemic bringing recruitment to an end and is underpowered. The control arm consisted of a treatment-as-usual intervention that was specific to each site, as such there is variability across the sites for those in the control group. A standardised active control condition would have controlled for any potential confounding factors due to site-specific treatment-as-usual therapies. Although all therapists were trained using the standard manuals for the treatments, and continued supervision provided, therapy sessions were not recorded and monitored for treatment fidelity. Our study identified changes in function using the SOFAS which is a crude measure of function in the community. Further work would benefit from the use of a more reliable and valid measure such as the UCSD Performance-based Skills Assessment which has been noted to have a better correlation with cognitive performance (43). Nonetheless, the study had strengths in that it was a multi-site investigation, run in standard youth mental health teams, supporting the clinical utility of the interventions. All assessments were blinded to treatment allocation. Participants represented a group of subjects under-represented in the literature, which has concentrated on older, more chronically unwell subjects. In addition, participants were followed up enabling us to observe if changes were maintained after the end of treatment.

In conclusion, this study suggests that there are advantages for community functioning in combining neurocognitive with social cognitive remediation therapy. The provision of treatment would be assisted by a more concentrated and intense treatment program that delivered the therapy over a shorter period. The availability of computer assisted cognitive remediation is now being enhanced by the development of similar programs targeting social cognition. We look forward to combining these programs and investigating the role of additional psychosocial interventions that are known to synergise the effects of CRT (8) to improve the outcomes for young people with SMI.

## Data Availability Statement

The raw data supporting the conclusions of this article will be made available by the authors without undue reservation.

## Ethics Statement

The studies involving human participants were reviewed and approved by Western Sydney Local Health District Human Research Ethics Committee. The patients/participants provided their written informed consent to participate in this study.

## Author Contributions

AH, CD, JW, and MK designed the proposal, recruited the participants, and contributed to the interpretation and writing of the paper. CM contributed to the analysis, interpretation, and writing of the paper. All authors contributed to the article and approved the submitted version.

## Funding

The study was funded by a Western Sydney Local Health District Research & Education Network Research Grant and by a Health Education and Training Institute Mental Health Research Award to JW. The funding sources had no input into any aspect of the study.

## Conflict of Interest

The authors declare that the research was conducted in the absence of any commercial or financial relationships that could be construed as a potential conflict of interest.

## Publisher's Note

All claims expressed in this article are solely those of the authors and do not necessarily represent those of their affiliated organizations, or those of the publisher, the editors and the reviewers. Any product that may be evaluated in this article, or claim that may be made by its manufacturer, is not guaranteed or endorsed by the publisher.
